# PEGylated liposome-encapsulated rhenium-188 radiopharmaceutical inhibits proliferation and epithelial–mesenchymal transition of human head and neck cancer cells in vivo with repeated therapy

**DOI:** 10.1038/s41420-018-0116-8

**Published:** 2018-10-31

**Authors:** Chun-Yuan Chang, Chao-Cheng Chen, Liang-Ting Lin, Chih-Hsien Chang, Liang-Cheng Chen, Hsin-Ell Wang, Te-Wei Lee, Yi-Jang Lee

**Affiliations:** 1 Department of Biomedical Imaging and Radiological Sciences, National Yang-Ming University, Taipei, Taiwan; 2 Department of Health Technology and Informatics, The Hong Kong Polytechnic University, Kowloon, Hong Kong; 3 Isotope Application Division, Institute of Nuclear Energy Research, Taoyuan, Taiwan; 4 Biophotonics and Molecular Imaging Research Center (BMIRC), National Yang-Ming University, Taipei, Taiwan

## Abstract

Human head and neck squamous cell carcinoma (HNSCC) is usually treated with chemoradiotherapy, but the therapeutic efficacy could be hampered by intrinsic radioresistance and early relapse. Repeated administrations of rhenium-188 (^188^Re)-conjugated radiopharmaceutical has been reported to escalate the radiation doses for better control of advanced human cancers. Here we found that high dosage of ^188^Re-liposome, the liposome-encapsulated ^188^Re nanoparticles exhibited significant killing effects on HNSCC FaDu cells and SAS cells but not on OECM-1 cells. To investigate the biological and pharmaceutical responses of high ^188^Re-liposomal dosage in vivo, repeated doses of ^188^Re-liposome was injected into the orthotopic tumor model. FaDu cells harboring luciferase reporter genes were implanted in the buccal positions of nude mice followed by intravenous injection of ^188^Re-liposome. The Cerenkov luminescence imaging (CLI) was performed to demonstrate an increased accumulation of ^188^Re-liposome in the tumor lesion of nude mice with repeated doses compared to a single dose. Repeated doses also enhanced tumor growth delay and elongated the survival of tumor-bearing mice. These observations were associated with significant loss of Ki-67 proliferative marker and epithelial–mesenchymal transition (EMT) markers in excised tumor cells. The body weights of mice were not significantly changed using different doses of ^188^Re-liposome, yet repeated doses led to lower blood counts than a single dose. Furthermore, the pharmacokinetic analysis showed that the internal circulation of repeated ^188^Re-liposomal therapy was elongated. The biodistribution analysis also demonstrated that accumulations of ^188^Re-liposome in tumor lesions and bone marrow were increased using repeated doses. The absorbed dose of repeated doses over a single dose was about twofold estimated for a 1 g tumor. Together, these data suggest that the radiopharmacotherapy of ^188^Re-liposome can enhance tumor suppression, survival extension, and internal circulation without acute toxicity using repeated administrations.

## Introduction

The incidence of head and neck squamous cell carcinoma (HNSCC) ranks the sixth most common human cancer globally, and over 600,000 cases are newly diagnosed annually^1^. HNSCC has high mortal rate (~350,000 death each year) because of sound recurrent and metastatic rates^2,3^. Additionally, surgical treatment or histological diagnosis of local invasion of human HNSCC usually leads to severe side effects including anatomic destruction, dysphasia, aphonia, and aphasia, which are caused by tumorous distribution around important physiological structures such as the spinal cord and carotid artery^4,5^. The intrinsic radioresistance is also related to the recurrence of HNSCC after chemoradiotherapy^6,7^. As adjuvant radiotherapy and chemotherapy remain a primary option for the treatment of HNSCC, development of optimal approaches for improvement of the therapeutic efficacy of HNSCC and maintenance of life quality is critical.

Rhenium-188 (^188^Re) is a high-energy β-particle radionuclide (2.12 MeV) with 15% γ-rays (155 keV) obtained from an alumina-based ^188^W/^188^Re generator^8^. The short average penetration distance of β-particles (around 3.8 mm) in soft tissues endows ^188^Re as an ideal radionuclide for tumor ablation, including the palliative therapy of bone metastasis with minimal harmful effects to surrounding normal tissues^9,10^. Polyethylene glycol (PEG)-decorated ^188^Re-liposome is a nano-sized biocompatible radiopharmaceutical that has been used for evaluating the theranostic efficacy in different human cancers, including colorectal cancer, glioblastomas, lung cancer, ovarian cancer, and esophageal cancer preclinically^11–15^. We have shown that ^188^Re-liposome could be accumulated in orthotopic HNSCC tumor lesions, but the therapeutic efficacy was moderate^16^. Radioresistance is a feature of HNSCC and is related to the tumor relapses after chemoradiotherapy^6^. Modification of treatment regime may be important to improve the therapeutic efficacy of ^188^Re-liposome.

Dose escalation of ^188^Re-conjugated radiopharmaceutical has been used for treatment of different human cancers. Palmedo et al.^17^ have found that an escalated dose of ^188^Re-HEDP (over 2.6GBq) offers 60–75% pain palliation in prostate cancer patients with osseous metastases with the occurrence of thrombocytopenia and leukopenia up to 8 weeks. Additionally, a perspective phase II clinical trial using 64 hormone-refractory prostate cancer patients concluded that enhanced pain palliation, reduced prostatic specific antigen (PSA), increased progression-free, and overall survival when patients received double injections rather than a single injection of ^188^Re-HEDP^18^. Repeated intratumoral injection of ^188^Re microspheres into the hepatoma animal model also achieves better therapeutic efficacy^19^. Whether repeated doses of ^188^Re-liposome can also enhance the therapeutic efficacy in HNSCC is of interest to study.

Epithelial–mesenchymal transition (EMT) is an important process of tumor metastasis. During EMT, epithelial cells can transit to mesenchymal phenotypes accompanied by high motility, which is caused by a loss of junction, cytoskeletal reorganization, and morphological change^20^. Such a transition is associated with vigorous reprogramming of gene expression, including E-cadherin, vimentin, zinc-finger E-box-binding 1 (ZEB-1), basic Helix-Loop-Helix Transcription Factor 1 (TWIST1), and Zinc-finger protein SNAI1 (SNAIL) transcription factors^21^. Intraperitoneal injection of ^188^Re-liposome has recently been reported to block EMT and reactivate p53 function in ovarian tumors^22^. A recent report showed that ^188^Re-liposome could induce the expression of *let-7* microRNA in HNSCC orthotopic tumors^16^. *Let-7* is known to inhibit EMT by suppressing the high-mobility group AT-hook 2 (HMGA2) gene that activates the expression of SNAIL and TWIST to inhibit tumor growth and metastasis^23–25^. Whether ^188^Re-liposome also influences the expression of EMT-related markers in HNSCC is of interest to investigate.

In this study, we showed that high doses of ^188^Re-liposome exhibited different killing efficacies on cultured HNSCC cells. To compare the low dose and high dose of ^188^Re-liposome on the therapeutic efficacy of HNSCC in vivo, we used a single therapy and repeated therapy to assess the biological and pharmaceutical responses in an orthotopic tumor model. Additionally, the systemic toxicity and markers of tumor proliferation and EMT, as well as dosimetry were examined and compared in tumor lesions treated with different dosages of ^188^Re-liposome. The significance of ^188^Re-liposome-based radiopharmacotherapy was discussed.

## Results

### Killing effects of ^188^Re-liposome on different HNSCC cell lines

**Fig. 1:**
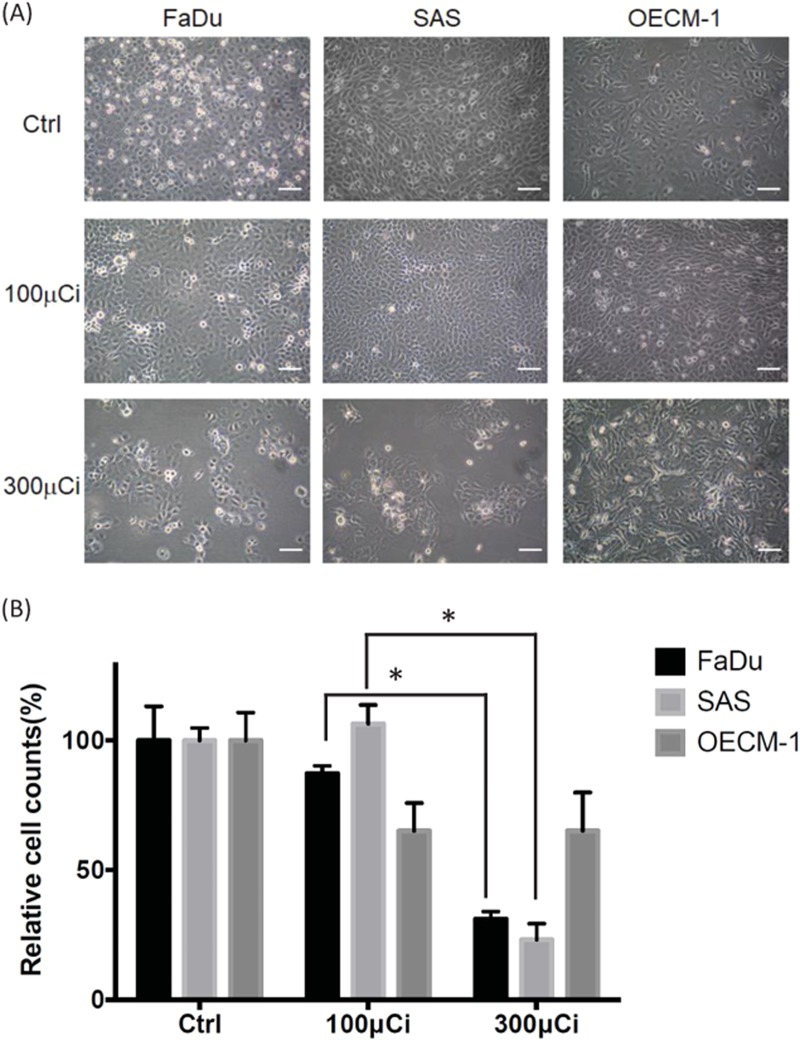
Effects of ^188^Re-liposome on different HNSCC cell lines using low dose and high dose. **a** Change of cell morphology and amount in FaDu cells, SAS cells, and OECM-1 cells treated with low dose (100 μCi) and high dose (300 μCi) of ^188^Re-liposome. Scale bar: 100 μm. **b** Quantification of cell number in cells treated with low or high dose of ^188^Re-liposome. Data were represented as means ± S.D. *p < 0.05 by a *t*-test

HNSCC includes malignancies initiating from different locations of the oral cavity. Here we administrated ^188^Re-liposome on three human HNSCC cell lines, including FaDu cells, SAS cells, and OECM-1 cells to examine the killing effects using different doses. It was found that cell killings of FaDu cells and SAS cells were more significant than that of OECM-1 cells using high dose (300 μCi) of ^188^Re-liposome (Fig. 1a, b). This is an important implication for clinical application of ^188^Re-liposome on different types of human HNSCC.

### Effects of single dose and repeated doses of ^188^Re-liposome on tumor targeting

**Fig. 2:**
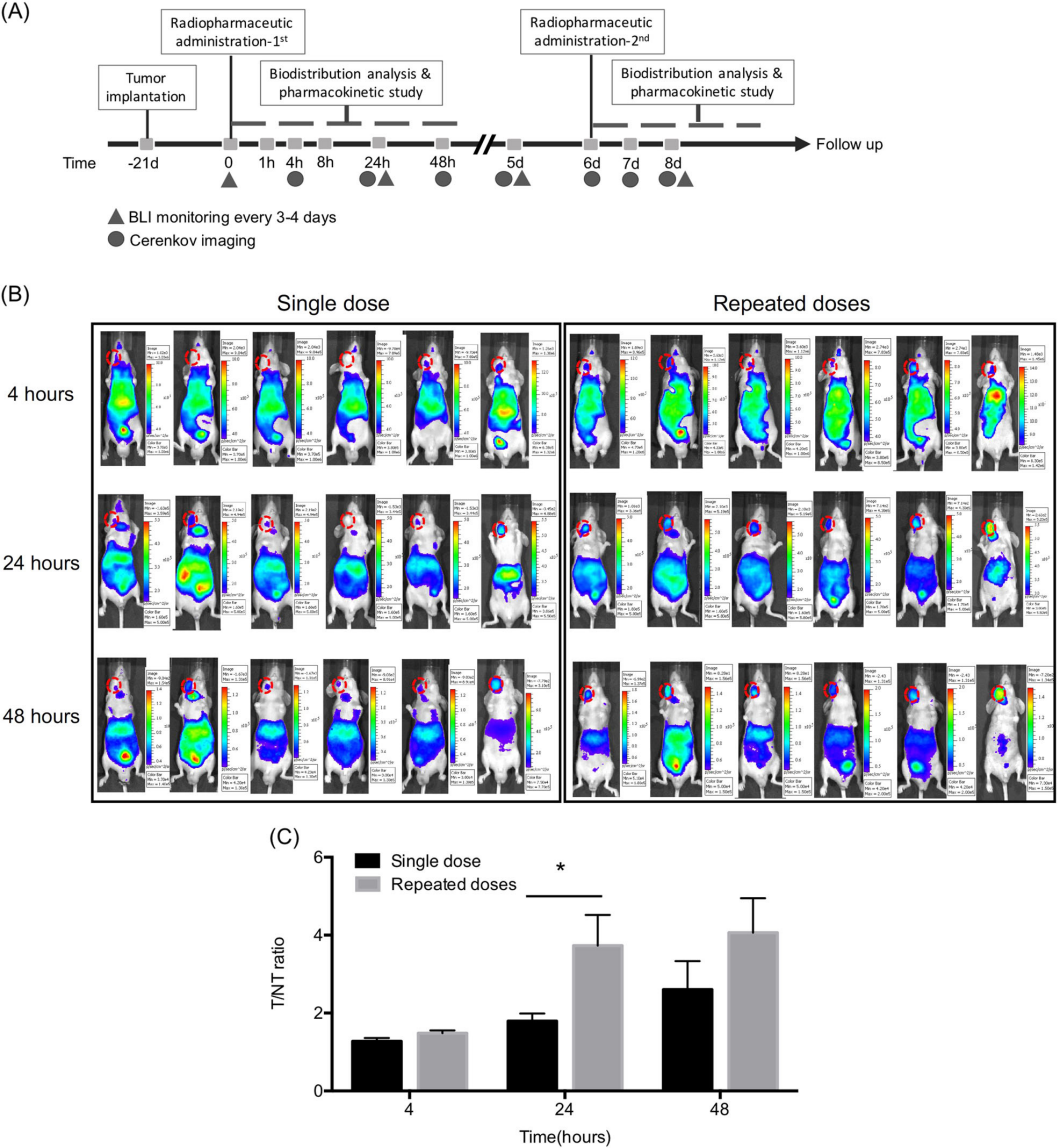
Comparison of PEGylated ^188^Re-liposomal accumulation in orthotopic HNSCC tumors after a single and repeated injections. **a** The experimental scheme for ^188^Re-liposome treatment. **b** CLI signals acquired by the IVIS system. **c** The ratios of photon flux were determined by normalizing the tumor (left side of mouth) to non-tumor (right side of mouth) regions. Red circles represented the region of interest (ROI) for tumor lesions (*n* = 6). Data were represented as means ± S.E.M. *p < 0.05 by a *t*-test

To investigate the response of HNSCC to ^188^Re-liposome in vivo, we established an orthotopic tumor model in immune-deficient nude mice using FaDu cells. The experimental regimes of a single i.v. injection or repeated i.v. injections of ^188^Re-liposome into tumor-bearing mice were schemed (Fig. 2a). The time interval for repeated injections of ^188^Re-liposome was 6 days as considered for the half-life of ^188^Re and clinical feasibility. Cerenkov luminescent imaging (CLI), an optical signal raised by charged particles using medical isotopes^26^, was firstly used to compare the ratios of ^188^Re-liposome accumulation in tumor-bearing mice between a single dose and repeated doses. Implantation of orthotopic tumor into the buccal position of each mouse exhibited a time-dependent increase of CLI signals after injection of ^188^Re-liposome (Fig. 2b). The signal intensity of each tumor lesion was normalized to a non-tumor region of the same tumor-bearing mouse. The results showed that repeated doses of ^188^Re-liposome tended to exhibit higher tumor-to-non-tumor ratio than a single dose of ^188^Re-liposome up to 48 h (Fig. 2c). The luminescent signals substantially disappeared right before the second injection (6 days after the first injection) of ^188^Re-liposome (Supplementary Data 1).

### Comparison of therapeutic efficacy between single injection and repeated treatment of ^188^Re-liposome on HNSCC animal model

**Fig. 3:**
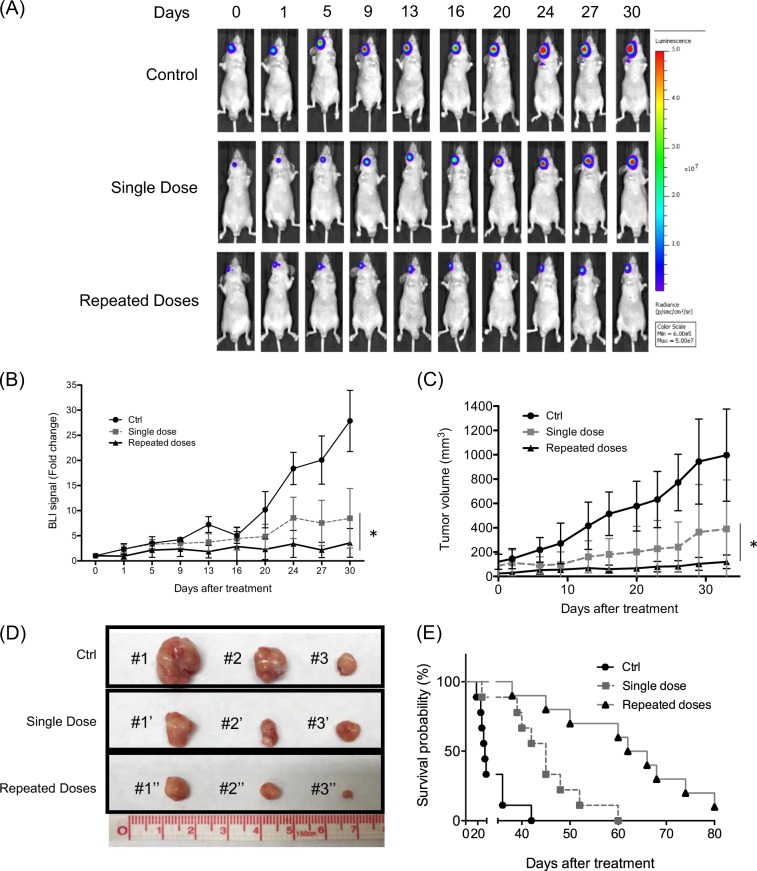
Monitoring the therapeutic efficacy of ^188^Re-liposome administrated in HNSCC tumor-bearing mice with different regimes. **a** Reporter gene imaging of tumor growth responding to a single dose, repeated doses of ^188^Re-liposome, and untreated control (*n* = 5). **b** Quantification of BLI signals. **c** Caliper measurement of tumor volumes (*n* = 5). Data were represented as means ± S.D. *p < 0.05 by an unpaired two-tailed *t*-test. **d** Representative photos of excised orthotropic tumors. **e** Analysis of animal survival using the Kaplan–Meier method with log-rank test (*p* < 0.001)

The therapeutic efficacy of ^188^Re-liposome was subsequently investigated using the bioluminescent imaging in FaDu-3R tumors that expressed luciferase activity. Repeated injections of ^188^Re-liposome exhibited better tumor suppressive effects than a single injection of ^188^Re-liposome in tumor-bearing mice (Fig. 3a). The results were also quantified by measuring the photon fluxes in untreated controls, a single dose, and repeated doses of ^188^Re-liposome (Fig. 3b). Additionally, repeated doses of ^188^Re-liposome exhibited slower tumor growth rates than a single dose and untreated control using caliper measurement of tumor volumes (Fig. 3c). The orthotopic tumors were also excised from tumor-bearing mice after 4 weeks of tumor growth to demonstrate the enhanced tumor suppressive effects by repeated doses of ^188^Re-liposome (Fig. 3d). Furthermore, the animal survival of repeated ^188^Re-liposome treatment was greater than that of a single ^188^Re-liposome treatment and untreated controls (Fig. 3e). The median survival times of tumor-bearing mice with repeated injections, a single injection, and untreated control were 64, 45, and 32 days, respectively.

### Effects of ^188^Re-liposome on expression of markers for proliferation and EMT using a single dose or repeated doses

**Fig. 4:**
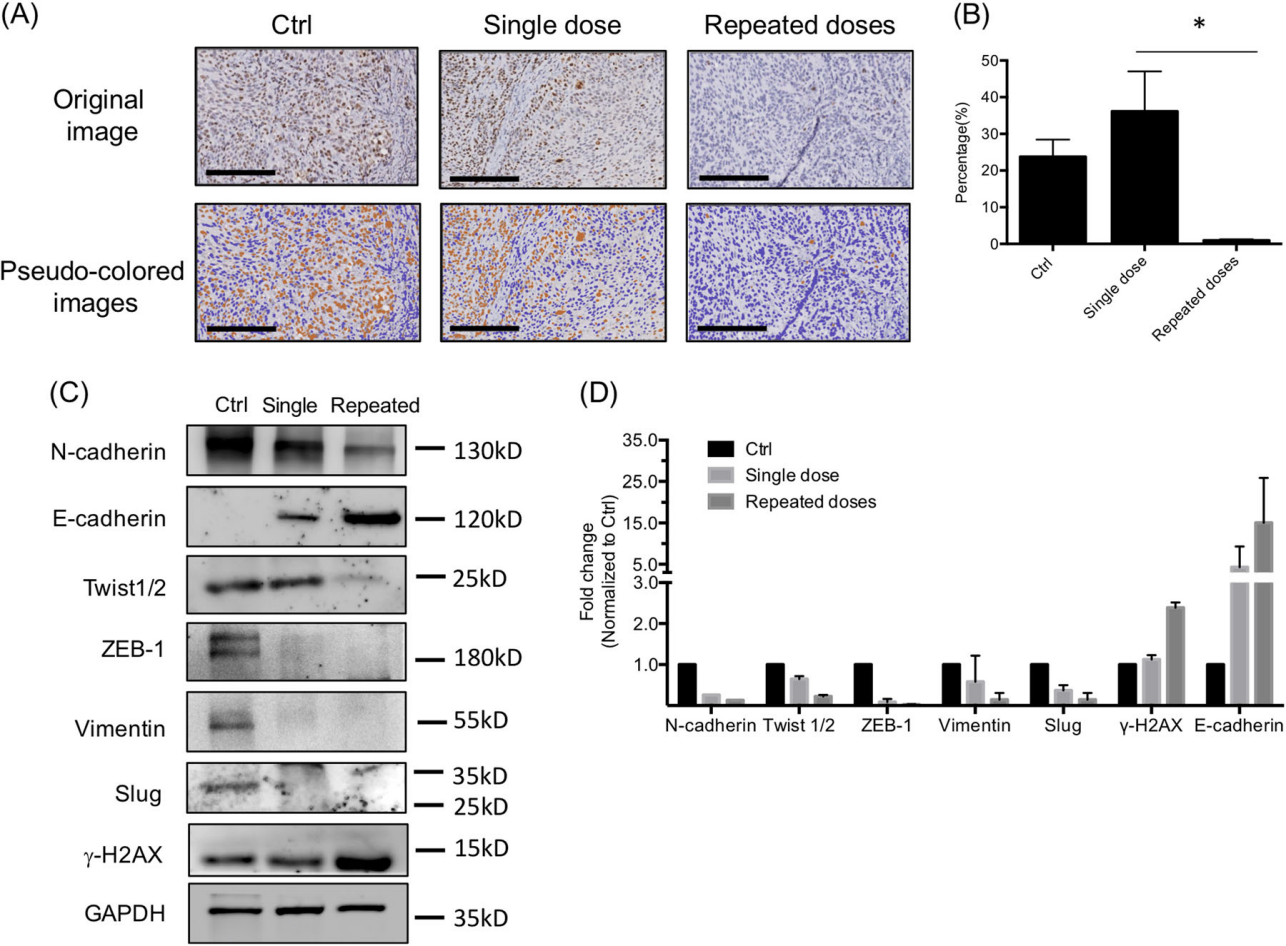
Effects of ^188^Re-liposome on the expression of molecular markers involved in cell proliferation and metastasis. **a** Measurement of IHC stained Ki-67 expression in orthotopic tumors. Scale bar, 200 μm. **b** Quantification of Ki-67 positive cells according to the images of pseudo-colored analysis. Three fields were randomly selected for counting the Ki-67 expressing cells. Data were represented as means ± S.D. *p < 0.05 by a *t*-test. **c** The expression of EMT-related markers in orthotopic tumors with different treatments of ^188^Re-liposome for 4 weeks. **d** Quantification of EMT-related markers detected in the blots using densitometry (*n* = 3). Data were represented as means ± S.D.

Tumors were also resected for IHC staining of Ki-67 proliferative marker. The level of Ki-67 was significantly suppressed by repeated doses of ^188^Re-liposome compared to a single dose and untreated controls demonstrated by the pseudo-colored image method (Fig. 4a, b). We also examined whether the expressions of EMT-related markers in tumors were affected by injection of ^188^Re-liposome. Compared to a single dose of ^188^Re-liposome, repeated dose apparently induced E-cadherin levels, inhibited N-cadherin and Twist1/2 levels (Fig. 4c). ^188^Re-liposome could equally suppress the levels of ZEB-1, vimentin, and Slug markers using a single dose or repeated dose (Fig. 4c). Interestingly, we also found that the level of γ-H2AX, a DNA damage marker was increased by repeated doses of ^188^Re-liposome compared to a single dose (Fig. 4c). These blots were also quantified using dosimetry (Fig. 4d).

### Comparison of toxic effects in tumor-bearing mice treated with a single dose and repeated doses of ^188^Re-liposome

**Fig. 5:**
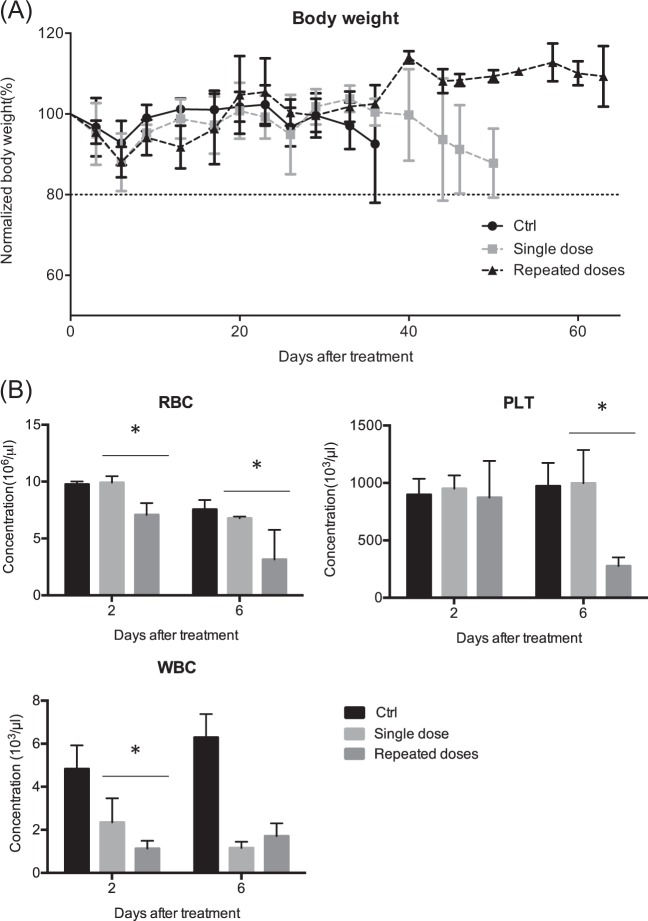
Evaluation of toxicity caused by single dose and repeated doses of ^188^Re-liposomal administrated in tumor-bearing mice. **a** Measurement of body weights (*n* = 5). The data point of each curve represented the mean ± S.D. of body weights averaged from five mice. **b** Counting of RBC, PLT, and WBC. The blood was obtained after mice were treated with a single dose or repeated doses of ^188^Re-liposome for 2 days and 6 days (*n* = 3). Data were represented as means ± S.D. *p < 0.05 by a *t-*test

We also compared the potent adverse effects in tumor-bearing mice treated with a single dose or repeated doses of ^188^Re-liposome. The changes of body weights were not significantly different by comparing the untreated controls and ^188^Re-liposome-injected groups (Fig. 5a). Moreover, compared to a single dose of ^188^Re-liposome, the counts of WBC, RBC, and platelets were significantly suppressed by repeated doses at different time points (Fig. 5b). A single injection of ^188^Re-liposome could also suppress the counts of WBC but not RBC and platelets.

### Comparison of biodistribution and pharmacokinetics in tumor-bearing mice treated with a single dose and repeated doses of ^188^Re-liposome

**Fig. 6:**
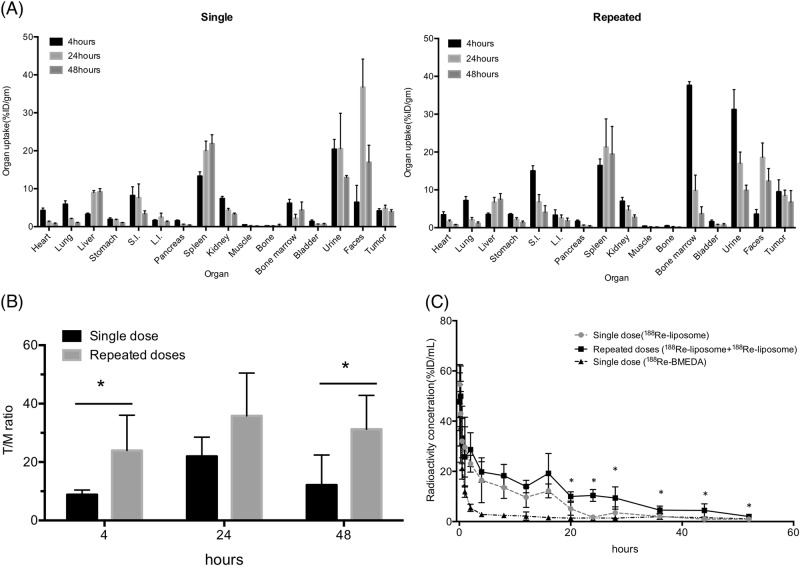
Biodistribution and pharmacokinetic analysis for comparing a single dose and repeated doses of ^188^Re-liposomal injection. **a** Biodistribution of single dose and repeated doses. (*n* = 5). S.I. small intestine, L.I. large intestine. **b** Comparison of tumor-to-muscle ratios between a single dose and repeated doses of ^188^Re-liposomal injection at different time points. **c** Pharmacokinetic analysis. (*n* = 5). Data were represented as means ± S.D. *p < 0.05 by a *t-*test

Biodistribution analysis was performed in two groups of mice injected with a single dose or repeated doses of ^188^Re-liposome. Compared to other organs, repeated doses exhibited higher accumulation rates of ^188^Re-liposome in bone marrows and tumors than a single dose (Fig. 6a and Supplementary Data 2). Moreover, the tumor-to-muscle ratio (T/M) of repeated doses to a single dose of ^188^Re-liposome was about twofold calculated from the biodistribution data up to 48 h (Fig. 6b). In regards of pharmacokinetics, liposome-free ^188^Re-BMEDA (640 μCi/150 μL) was used as a control to compare the circulation of ^188^Re-liposome using a single injection or repeated injections into mice. The results showed that the injection of ^188^Re-liposome exhibited slower clearance and longer retention than ^188^Re-BMEDA, and these effects were even greater in repeated doses than in a single dose of ^188^Re-liposome (Fig. 6c). The pharmacokinetic-related parameters were compared between intravenous injections of ^188^Re-BMEDA and ^188^Re-liposome (Table 1). Notably, the AUC of repeated doses was 1.65-fold to that of a single dose.

**Table 1:** Pharmacokinetic analysis for single dose and repeated dose of ^188^Re-liposome treatment on HNSCC tumor-bearing mice

Parameter	Unit	Single (^188^Re-BMEDA)	Single (^188^Re-liposome)	Repeated (^188^Re-liposome)
*C*_max_	%ID/mL	43.51 ± 10.16	55.37 ± 7.04	54.99 ± 8.48
Cl	mL/h	1.09 ± 0.20	0.31 ± 0.12	0.17 ± 0.04*
AUC_(0→∞)_	h ·[%ID/mL]	74.71 ± 10.46	348.75 ± 119.54	585.72 ± 141.29*
MRT	h	5.38 ± 0.50	11.55 ± 1.39	16.52 ± 1.72**

For PK analysis in this table, data represent means ± SD, *p < 0.05, **p < 0.01

*C*_*max*_ : Larger maximal concentration, *%ID* : percentage injected dose, *Cl:* clearance, *AUC(0*_*→∞*_*)* : area under curve, *MRT(0*_*→∞*_*):* mean residence time [*Please change MRT(0->∞) to MRT]

### Dosimetric analysis for a single dose and repeated doses of ^188^Re-liposome administrating in the HNSCC tumor model

The estimated internal radiation doses were compared between a single dose and repeated doses of ^188^Re-liposome (Supplementary Data 3). Compared to a single dose of ^188^Re-liposome, repeated doses caused over twofold absorbed dose in the bladder wall (0.497 verses 0.0891mGy/MBq), red marrow (0.395 verses 0.145 mGy/MBq), and small intestine (0.377 verses 0.0899mGy/MBq). The effective doses were 0.177 and 0.245 mSv/MBq for a single dose and repeated doses of ^188^Re-liposome, respectively. The estimated tumor absorbed doses of a single dose and repeated doses were 0.136 and 0.264 mGy/MBq using a 1 g spheroid model, respectively. The absorbed doses for different sizes of spheroid tumor mass treated with ^188^Re-liposome with a single dose or repeated doses were also performed (Supplementary Data 4).

## Discussion

HNSCC includes different cell types in head and neck regions that exhibit heterogeneous responses to radiation therapy. Here we used high dose of ^188^Re-liposome to treat three different HNSCC cell lines, and demonstrated that significant cell killing was only found in FaDu cells and SAS cells but not in OECM-1 cells. Despite FaDu cells were further used for the establishment of orthotopic tumor model, OECM-1 cells were also attempted to be implanted into nude mice. However, this cell type failed to form tumors (data not shown). This is consistent with a previous report that OECM-1 tumor is barely formed^27^. Little is known about the mechanisms of ^188^Re-liposome mediated cell death. It has been reported that mutation of p53, retinoblastoma (pRb), NOTCH, phosphoinositol-3 kinase (PI3K), phosphatase and tensin homolog (PTEN), AKT kinase, and epithelial growth factor receptor (EGFR) pathways are commonly found in the occurrence of HNSCC^28^. Interestingly, targeting survivin also induce both apoptotic and autophagic cell death in HNSCC^29^. Whether different cell killing efficacies mediated by ^188^Re-liposome is associated with these pathways will be important to further investigate.

^188^Re is a cost-effective isotope with theranostic potent^30^. Due to its affordable price, a dose-escalation study using ^188^Re-HEDP has been reported to evaluate its effects on pain palliation of prostate cancer patients with osseous metastases^17^. However, a phase I dose-escalation trial has been used to determine the dose-limiting toxicity (DLT), and the suppression of red marrow was the only DLT to be observed^31^. Administration of relatively high doses of ^188^Re may be a potent candidate for radioimmunotherapy, although increased renal and liver uptakes were also detected^31^. Additionally, the short physical half-life of ^188^Re (*T*_1/2_ = 16.9 h) is an important property for repeated treatment^32^. ^188^Re-HEDP has been applied for pain relief therapy of bone metastases secondary to breast cancer and prostate cancer^33^. The results of clinical trials suggest that the use of ^188^Re-conjugated radiopharmaceuticals with repeated doses for cancer treatment should be feasible. Using animal models, it is possible to compare the effects of a single dose and repeated doses of ^188^Re-conjugated radiopharmaceutical on various human cancers for clinical consideration. Here we used the radioresistant FaDu cancer cells^34^ to demonstrate that repeated therapy of ^188^Re-liposome was more effective than a single therapy in the suppression of tumors formed by these cells. Repeated treatments with original doses rather than an escalated single dose were adopted to avoid exceeding 80% MTD. These preclinical results are partially consistent with previous reports that repeated treatments of ^188^Re-conjugated radiopharmaceutical will provide better tumor control in clinical trials^18,35^.

The improved therapeutic effects of repeated doses of ^188^Re-liposome can be elucidated by the tumor accumulation of this radiopharmaceutical and the molecular responses of the orthotopic tumor. The hypopharyngeal cancer FaDu cells can induce angiogenesis in nude mice^36^. The enhanced permeability and retention (EPR) effect should not be impaired by the first injection of repeated ^188^Re-liposome doses as both single injection and repeated injections of ^188^Re-liposome could accumulate in tumor lesions. Interestingly, repeated doses of ^188^Re-liposome could change the expression of several EMT-related molecules. A recent report showed that ^188^Re-liposome could induce E-cadherin and suppress vimentin in human ovarian cancer cells^22^. In this study, this effect was further demonstrated in an HNSCC tumor model by examining additional EMT-related markers, which were also enhanced by repeated doses of ^188^Re-liposome. Notably, we found that the level of γ-H2AX, a DNA double-strand breaks marker was also higher in tumors treated with repeated doses of ^188^Re-liposome. It has been reported that ZEB-1 could promote DNA repair and lead to radioresistance in cancer cells^37^. Hence, suppression of ZEB-1 by repeated doses of ^188^Re-liposome may suppress the DNA repair effects and increase the radiosensitivity of orthotopic tumors. As EMT may have a pivotal role in the recurrence of HNSCC^38^, inhibition of EMT by repeated therapy of ^188^Re-liposome may also reduce the probability of recurrence.

The side effects were the primary concerns when the repeated therapy of ^188^Re-liposome was adopted. It is assumed that optimal time interval between the first dose and second dose may reduce potent toxicity without loss of therapeutic efficacy. In this study, the time interval of repeated ^188^Re-liposomal injections was 6 days (approximately over 9 half-lives of decay). No significant reduction of body weight was detected after repeated therapy; therefore, this treatment should not cause acute toxicity. On the other hand, the counts of RBC, WBC, and PLT were suppressed by ^188^Re-liposome; and repeated doses exhibited stronger effects than a single dose. Hence, repeated doses of ^188^Re-liposome may increase the possibility of hematologic impairment. These results were partially consistent with ^188^Re-HEDP that showed clinically unimportant decreases in WBC and platelet using repeated doses of ^186^Re-HEDP with a time interval at 8–12 weeks^39^.

The circulation period of repeated ^188^Re-liposomal doses was longer than that of a single dose as shown by pharmacokinetic analysis, suggesting that the enhanced therapeutic efficacy is associated with longer retention of ^188^Re-liposome after repeated injections. On the other hand, it may imply that elongated circulation of ^188^Re-liposome increases bone marrow dose and reduces blood counts. It is consistent with a previous report that bone marrow toxicity was a main limiting factor of ^186^Re-HEDP^40^, at least in part.

The OLINDA/EXA code based dosimetric calculation revealed that the bladder wall, small intestine, and red marrow received over twofold of absorbed dose after repeated therapy of ^188^Re-liposome. The ratios of enhancement were 5.58, 4.19, and 2.72 for the bladder wall, small intestine, and red marrow, respectively. As the tolerance doses of the bladder wall (50–70 Gy) and small intestine (20–45 Gy) are higher than for red marrow (2–10 Gy) in radiotherapy^41,42^, using repeated doses of ^188^Re-liposome may be acceptable for clinical purposes. The effective dose of both single dose and repeated doses calculated for a male adult model remains far lower than a single diagnostic procedure using radionuclide (1–10 mSv) or background radiation amount (about 3 mSv/year)^43^.

In summary, current data indicate that in cultured cells, high dose of ^188^Re-liposome would perform various killing effects on different origins of HNSCC cells. For in vivo study, repeated doses of ^188^Re-liposome exhibited greater tumor ablation and survival than a single dose administrated in the HNSCC tumor model. Extended circulation time of ^188^Re-liposome might contribute to increased accumulation of this radiopharmaceutical in tumor lesions after repeated administration to enhance the tumor suppression. Although acute toxicity was not detected, a significant decrease of blood cells could be a limiting factor when using repeated therapy of ^188^Re-liposome for HNSCC treatment. Good hospitalization or prevention of immunological/hematological impairment should be considered for repeated therapy of ^188^Re-liposome in clinical application.

## Materials and methods

### Cell lines, plasmid, and cell counts

Human FaDu hypopharyngeal carcinoma cells (American Type Culture Collection, Manassas, VA, USA) and FaDu-3R cells harboring a pLT-3R construct with multiple reporter genes were maintained as a previous report^16^. Cells were maintained in RPMI-1640 (Life Technologies Inc., Carlsbad, CA, USA) medium. Human tongue carcinoma SAS cell line was a kind gift obtained from Prof. Muh-Hua Yang (National Yang-Ming University, Taipei, Taiwan) and was cultured in Dulbecco’s modified Eagle’s medium (DMEM). Oral squamous cell carcinoma OECM-1 was a kind gift from Dr. Yu-Jen Chen (Department of Radiation Oncology, MacKay Memorial Hospital, Taipei, Taiwan) and was cultured in RPMI-1640. All cell lines were supplemented with 10% fetal bovine serum (FBS), 2 mM l-glutamine, 50 U/mL of penicillin and 50 μg/mL of streptomycin (Invitrogen Inc., Carlsbad, CA), and were incubated at 37 °C in a humidified incubator with 5% CO_2_ and passaged every two days. Cell counts were also used for evaluation of cell viability before and after drug treatment. Briefly, cells (5 × 10^5^) were seeded in 10-cm dishes and incubated overnight. The medium was then replaced by fresh medium containing different concentrations of ^188^Re-liposome. Cell images were acquired after cells were exposed to ^188^Re-liposome for 72 h, and cell numbers were counted using hemocytometry.

### Preparation of ^188^Re-liposome for intravenous injection

The procedures of ^188^Re-liposome preparation of validation have been described before^11^. The mean loading efficiency of ^188^Re-liposome was ~70–80% determined by (Total radioactivity eluted)/(Remnant radioactivity in chromatographic column). Each injection used 23.68MBq (640 μCi) corresponding to 80% maximum tolerated dose (MTD) as described previously^14^.

### Establishment of HNSCC orthotopic tumor model

The orthotopic implantation of FaDu-3R cells in BALB/c nude mice has been described previously^16^. Animal experiments had been approved by the Institutional Animal Care and Utilization Committee (IACUC) of National Yang-Ming University (No. 1041106).

### Evaluation of tumor uptake and therapeutic efficacy of ^188^Re-liposome in tumor-bearing mice

After administration of ^188^Re-liposome, CLI was performed to acquire signals using the In Vivo Imaging System (IVIS 50, Perkin Elmer Inc., Waltham, MA, USA). For evaluation of therapeutic efficacy, the tumor viability and growth rate were assessed using the luciferase based reporter gene imaging and caliper measurement, respectively. The tumor volume was determined by the formula: (width^2^ × length)/2 after caliper measurement. For survival analysis, the end point of each datum was established when tumor volume reached 1000 mm^3^ by caliper measurement, or when the body weight reduced over 25% from the first day of treatment.

### Immunohistochemical (IHC) staining

The paraffin embedded tissue sections were prepared and incubated with anti-Ki-67 antibody (MAB4190, EMD Millipore, Billerica, MA, USA) at 4 °C overnight followed by horseradish peroxidase (HRP)-conjugated secondary antibodies. All sections were scanned by the Aperio digital Pathology Slide Scanner (Leica Biosystems, Buffalo Grove, IL, USA). The images were subjected to the ImmunoRatio automated counting tool to estimate the Ki-67 positivity index of the nuclei^44^.

### Western blot analysis and antibodies

Tumors were harvested from the tumor-bearing mice after 4 weeks of treatment and then lysed in T-PER™ Tissue Protein Extraction Reagent (Thermo Fisher Scientific, Waltham, MA, USA) containing 1% protease inhibitor cocktail (Sigma-Aldrich Co., St. Louis, MO, USA). The procedures of Western blot analysis have been reported previously^45^. The primary antibodies used in this study included anti-N-cadherin (GTX100443), anti-E-cadherin (GTX100443), anti-Twist1/2 (GTX127310), anti-ZEB-1 (GTX105278), anti-vimentin (GTX100619), anti-Slug (GTX128796), anti−γ-H2AX (GTX628789; GeneTex, Inc., Irvine, CA, USA), and anti-GAPDH (MA5-15738; Thermo Fisher Scientific, Waltham, MA, USA).

### Measurement of blood cell counts

Blood samples were acquired from mice at different time points after the treatment of ^188^Re-liposome by orbital sinus sampling. Red blood cells (RBC), white blood cells (WBC), and platelets were recorded by XT-1800i, an automated hematology analyzer (Sysmex Co., Chuo-ku, Kobe, Hyogo, Japan).

### Analysis of biodistribution and pharmacokinetic

The tumor-bearing mice were randomly assigned to three groups for injection of ^188^Re-liposome or ^188^Re-BMEDA followed by biodistribution and pharmacokinetic analysis as reported previously with slightly modification^16^. For biodistribution, mice were killed by CO_2_ asphyxiation after intravenous injection of ^188^Re-liposome followed by harvesting of different organs. Samples were weighted and counted by a γ-scintillation counter (1470 WIZARD Gamma Counter, Wallac, Finland). The results were represented as the percentage injected dose per gram tissue (% ID/g). For pharmacokinetic analysis, the blood samples were collected from mice using the tail vein puncture with microliter capillary tubes at 0.083, 025, 0.5, 1, 2, 4, 8, 12, 16, 20, 24, 28, 36, 44, and 52 h. Samples were then counted by a γ-scintillation counter and calculated by the WinNonLin software (v6.6, Pharsgiht Corp., Mountain View, California, USA) using a non-compartment model.

### Dosimetric evaluation of ^188^Re-liposomal absorbed dose in vivo

The dosimetry of percentage injected dose activity per weight tissue (%ID/g) in human was extrapolated from the biodistribution data of mice using the guideline of Medical Internal Radiation Dosimetry (MIRD) pamphlets implanted in the OLINDA/EXM software^46,47^. The number of disintegration of tumor was used to calculate the absorbed dose in tumor (1 g) using the sphere model.

### Statistical analysis

The statistical differences were analyzed by a two-tailed *t*-test (GraphPad Prism 6.0; GraphPad Software, San Diego, CA, USA). All data were represented as mean ± S.D. or mean ± S.E.M. Use of statistic methods and sample numbers were also described in each figure legend. The Kaplan–Meier method with the log-rank test was used to compare survival rates among different treatments. The level of statistical significance was set to *p* < 0.05 for all tests.

## Electronic supplementary material

Supplementary data 1

Supplementary data 2

Supplementary data 3

Supplementary data 4
