# Supplementary material for: PEGylated liposome-encapsulated rhenium-188 radiopharmaceutical inhibits proliferation and epithelial–mesenchymal transition of human head and neck cancer cells in vivo with repeated therapy
Source: Cell Death Discov. 2018 Oct 31;4:100. doi: 10.1038/s41420-018-0116-8 (PMC6208374; doi:10.1038/s41420-018-0116-8)
Supplement: Supplementary file 1 — Supplementary data 1 [file 41420_2018_116_MOESM1_ESM.pdf]

(A)

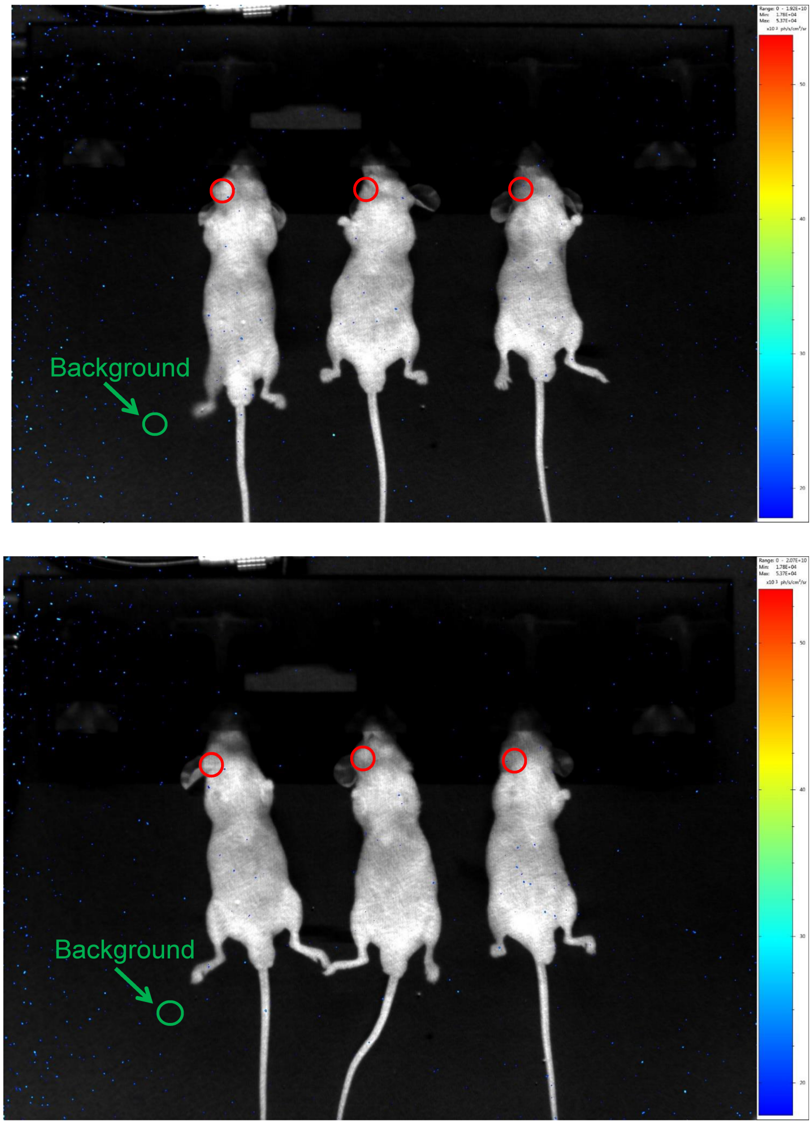

(B)

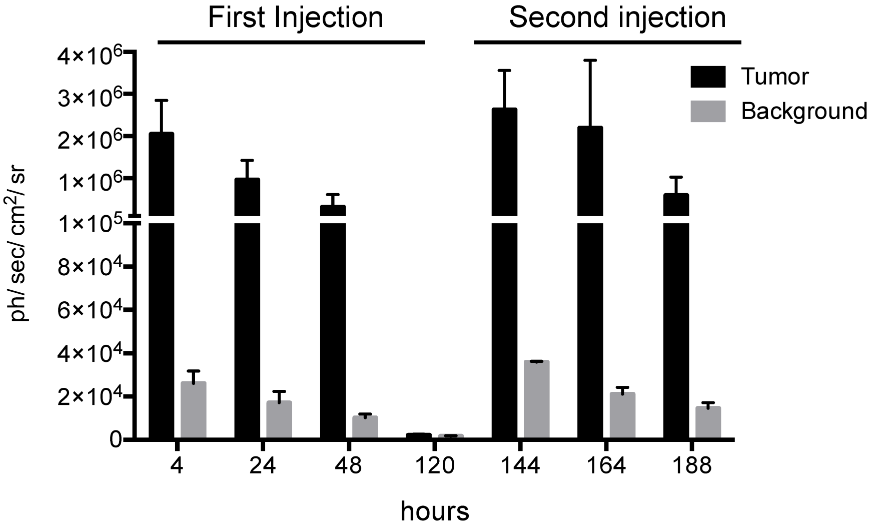

Supplementary Data 1. Change of Cerenkov luminescent signals between first injection and second injection of ^{188}Re-liposome to tumor-bearing mice. (A) The Cerenkov luminescent signals were disappeared after 120 hours of first injection of ^{188}Re-liposome; (B) time-course dependent change of Cerenkov luminescent signals.
