# Supplementary material for: PEGylated liposome-encapsulated rhenium-188 radiopharmaceutical inhibits proliferation and epithelial–mesenchymal transition of human head and neck cancer cells in vivo with repeated therapy
Source: Cell Death Discov. 2018 Oct 31;4:100. doi: 10.1038/s41420-018-0116-8 (PMC6208374; doi:10.1038/s41420-018-0116-8)
Supplement: Supplementary file 2 — Supplementary data 2 [file 41420_2018_116_MOESM2_ESM.pdf]

Supplementary Data 2: Radioactivity distribution in tumor and other organs of FaDu-3R tumor-bearing mice after intravenous injection of ^{188}Re-liposome.

% ID/g	Single dose			Repeated doses		
	4 hours	24 hours	48 hours	4 hours	24 hours	48 hours
Heart	4.27±0.64	1.26±0.20	0.75±0.23	3.43±0.78	1.62±0.40	0.74±0.15
Lung	5.90±0.91	2.06±0.13	0.98±0.16	7.18±1.05	2.08±0.61	1.13±0.41
Liver	3.27±0.32	8.95±0.56	9.19±0.84	3.50±0.39	6.71±1.29	7.50±1.52
Stomach	1.91±0.43	1.78±0.20	1.00±0.12	3.50±0.25	2.19±0.48	1.33±0.43
S.I.	8.19±2.32	7.63±3.62	3.38±0.80	15.03±1.36	6.78±1.99	4.05±1.77
L.I.	1.63±0.22	2.47±1.08	1.22±0.27	3.32±1.43	2.57±0.80	1.83±0.72
Pancreas	1.57±0.21	0.58±0.08	0.36±0.12	1.68±0.37	0.58±0.17	0.40±0.15
Spleen	13.31±1.14	20.00±2.54	21.85±2.36	16.43±1.74	21.29±7.46	19.45±7.33
Kidney	7.41±0.57	4.33±0.51	3.27±0.31	7.00±1.01	4.67±1.09	2.74±0.66
Muscle	0.47±0.06	0.23±0.05	0.14±0.06	0.41±0.07	0.21±0.06	0.16±0.06
Bone	0.20±0.04	0.20±0.04	0.33±0.31	0.48±0.13	0.26±0.07	0.14±0.05
Bone marrow	6.16±1.03	2.21±1.05	4.37±2.14	37.62±0.99	9.83±4.08	3.69±1.84
Bladder	1.38±0.39	0.62±0.11	0.61±0.26	1.59±0.44	0.74±0.18	0.72±0.38
Urine	20.40±2.60	20.53±9.34	12.95±0.51	31.24±5.24	16.99±3.00	9.87±1.38
Faces	6.42±4.45	36.75±7.42	16.96±4.50	3.59±1.21	18.56±3.84	12.33±3.31
Tumor	4.15±0.58	4.78±0.87	3.92±0.57	9.49±3.17	8.46±1.48	6.76±3.06
T/M ratio	8.86±1.57	21.97±6.54	12.15±10.2	23.97±12.07	35.82±14.63	31.23±11.59
