# Supplementary material for: PEGylated liposome-encapsulated rhenium-188 radiopharmaceutical inhibits proliferation and epithelial–mesenchymal transition of human head and neck cancer cells in vivo with repeated therapy
Source: Cell Death Discov. 2018 Oct 31;4:100. doi: 10.1038/s41420-018-0116-8 (PMC6208374; doi:10.1038/s41420-018-0116-8)
Supplement: Supplementary file 3 — Supplementary data 3 [file 41420_2018_116_MOESM3_ESM.pdf]

Supplementary Data 3: Dosimetric estimation of single dose and repeated doses of ^{188}Re-liposome in human organs extrapolated from animal data[a].

	Absorbed dose (mGy/MBq)	
Target Organ	Single dose	Repeated doses
Adrenals	8.98E-02	9.02E-02
Brain	8.87E-02	8.89E-02
Gallbladder Wall	9.03E-02	9.06E-02
LLI Wall[b]	8.93E-02	1.50E-01
Small Intestine	8.99E-02	3.77E-01
Stomach Wall	8.96E-02	8.98E-02
ULI Wall[b]	8.98E-02	9.10E-02
Heart Wall	3.95E-01	3.30E-01
Kidneys	2.07E-01	1.78E-01
Liver	2.79E-01	2.29E-01
Lungs	1.31E-01	1.51E-01
Muscle	1.44E-02	1.38E-02
Ovaries	8.95E-02	9.07E-02
Pancreas	3.86E-02	4.09E-02
Red Marrow	1.45E-01	3.95E-01
Osteogenic Cells	2.02E-01	3.61E-01
Skin	8.72E-02	8.73E-02
Spleen	6.98E-01	7.30E-01
Testes	8.81E-02	8.84E-02
Thymus	8.90E-02	8.91E-02
Thyroid	8.84E-02	8.86E-02
Urinary Bladder Wall	8.91E-02	4.97E-01
Uterus	8.96E-02	9.01E-02
Effective Dose(mSv/MBq)	1.77E-01	2.45E-01

a. The radiation dosimetry was converted from the biodistribution of ^{188}Re-liposome in a 0.025kg mouse to a 70kg male model. b. LLI: Lower Large Intestine; ULI: Upper Large intestine.
