# Supplementary material for: PEGylated liposome-encapsulated rhenium-188 radiopharmaceutical inhibits proliferation and epithelial–mesenchymal transition of human head and neck cancer cells in vivo with repeated therapy
Source: Cell Death Discov. 2018 Oct 31;4:100. doi: 10.1038/s41420-018-0116-8 (PMC6208374; doi:10.1038/s41420-018-0116-8)
Supplement: Supplementary file 4 — Supplementary data 4 [file 41420_2018_116_MOESM4_ESM.pdf]

Supplementary Data 4: Dosimetric estimation of absorbed dose in tumor sphere model.

Doses from Nuclide: Re-188 in Spheres:		
	Dose (mGy/MBq)	
Sphere Mass (g)	single	repeated
0.01	5.30E+00	1.03E+01
0.1	1.03E+00	2.01E+00
0.5	2.56E-01	4.98E-01
1	1.36E-01	2.64E-01
2	7.09E-02	1.38E-01
4	3.68E-02	7.15E-02
6	2.49E-02	4.85E-02
8	1.89E-02	3.68E-02
10	1.52E-02	2.95E-02
20	7.75E-03	1.51E-02
40	3.94E-03	7.67E-03
60	2.65E-03	5.16E-03
80	2.00E-03	3.89E-03
100	1.60E-03	3.11E-03
300	5.42E-04	1.05E-03
400	4.08E-04	7.93E-04
500	3.26E-04	6.35E-04
600	2.73E-04	5.31E-04
1000	1.65E-04	3.21E-04
2000	8.43E-05	1.64E-04
3000	5.63E-05	1.10E-04
4000	4.24E-05	8.24E-05
5000	3.40E-05	6.61E-05
6000	2.82E-05	5.50E-05
